# The Pathogenesis of Cytomegalovirus and Other Viruses Associated with Hearing Loss: Recent Updates

**DOI:** 10.3390/v15061385

**Published:** 2023-06-16

**Authors:** Xinyu Shi, Xiaozhou Liu, Yu Sun

**Affiliations:** 1 Department of Otorhinolaryngology, Union Hospital, Tongji Medical College, Huazhong University of Science and Technology, Wuhan 430022, China; sxy2017@hust.edu.cn (X.S.); d202181808@hust.edu.cn (X.L.); 2 Hubei Province Key Laboratory of Oral and Maxillofacial Development and Regeneration, Wuhan 430022, China; 3 Institute of Otorhinolaryngology, Union Hospital, Tongji Medical College, Huazhong University of Science and Technology, Wuhan 430022, China

**Keywords:** hearing loss, sensorineural hearing loss, virus, cytomegalovirus, SARS-CoV-2

## Abstract

Virus infection is one of the most common etiologies of hearing loss. Hearing loss associated with viral infection can be unilateral or bilateral, mild or severe, sudden or progressive, and permanent or recoverable. Many viruses cause hearing loss in adults and children; however, the pathogenesis of hearing loss caused by viral infection is not fully understood. This review describes cytomegalovirus, the most common virus causing hearing loss, and other reported hearing loss-related viruses. We hope to provide a detailed description of pathogenic characteristics and research progress on pathology, hearing phenotypes, possible associated mechanisms, treatment, and prevention measures. This review aims to provide diagnostic and treatment assistance to clinical workers.

## 1. Introduction

Hearing loss (HL) is one of the most common sensory disorders in humans. According to the World Hearing Report released by the World Health Organization (WHO), 1.5 billion people worldwide suffer from varying degrees of hearing loss [1]. Although hearing loss may cause mental retardation and psychological impairment in children, reduce employment opportunities for adults [2], and even accelerate the decline of cognitive ability in the elderly [3], the impact of hearing loss on patients is often overlooked. The etiology of hearing loss includes genetics, viruses, drugs, and environmental factors. Multiple viral infections have been shown to result in hearing loss. It is estimated that more than 30% of cases of hearing loss in children are caused by ear infections, such as measles, mumps, and rubella [1]. Due to large-scale outbreaks in recent years, the complications of viral infections, especially hearing loss, have received global attention.

**Figure 1:**
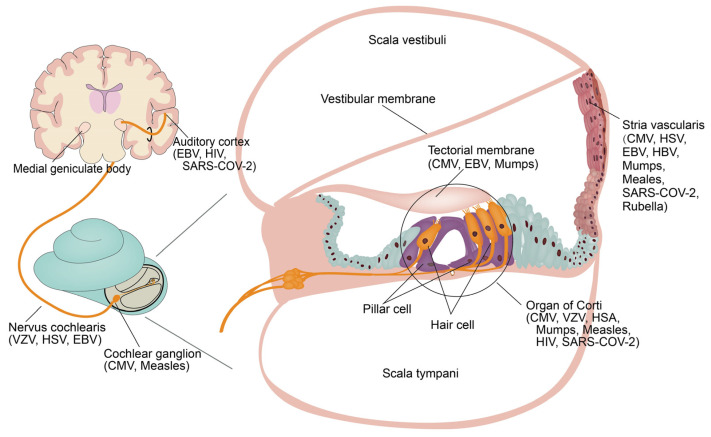
Schematic diagram of the infected area of the virus in the auditory pathway. In human or animal models, viruses may exist in different locations of the auditory pathway and play important roles in hearing loss directly or indirectly. CMV, EBV, and mumps may influence tectorial membrane; CMV, HSV, EBV, HBV, mumps, measles, SARS-CoV-2, and rubella may influence stria vascularis; CMV, VZV, HSA, mumps, measles, HIV, and SARS-CoV-2 may influence the organ of Corti; CMV and measles may influence the cochlear ganglion; VZV, HSV, and EBV may influence the nervus cochlearis; EBV, HIV, and SARS-CoV-2 may influence the auditory cortex.

Many viruses are responsible for congenital or acquired hearing loss; additionally, viruses may cause otosclerosis, and conductive hearing loss has also been reported (Table 1). Hearing loss caused by viruses can be mild, moderate, or even profound, unilateral or bilateral, and sudden or progressive [4]. Studies have shown that viral infection may directly damage the cells and structures of the inner ear (Figure 1); the inflammatory response triggered by viral infection may also be one of the causes of inner ear damage. The pathophysiology of virus-induced hearing loss remains incompletely understood, which creates difficulties in diagnosis and treatment.

In this review, we describe reported viruses related with hearing loss, along with their pathology, hearing phenotypes, possible associated mechanisms, treatment, and prevention measures, which may be helpful for researchers and clinical doctors to increase understanding about pathophysiological of virus-associated hearing loss.

## 2. Herpesviruses

### 2.1. Cytomegalovirus

Cytomegalovirus (CMV) is a member of the beta subfamily of the family *Herpesviridae*. The modes of transmission of CMV include vertical transmission [5], contact transmission [6], and sexual transmission [7]. Congenital CMV (cCMV) infection is the most common congenital viral infection [8], and CMV is one of the most common viruses that cause non-genetic SNHL (sensorineural hearing loss) and neurodevelopmental disorders, with CMV infections causing up to 10–20% of hearing loss among children [9]. In industrialized countries, the birth prevalence of cCMV ranges from 0.3% to 2.4%, while, in developing countries, this figure is likely to be higher [10]. The estimated CMV seroprevalence in women of childbearing age is 86% [10]. The risk of intrauterine transmission is highest when primary infection occurs during pregnancy, with a higher vertical transmission rate in mothers with an older gestational age at infection. Additionally, the risk of adverse fetal effects significantly increases if fetal infection occurs during the first half of pregnancy [8].

Adults or immune populations usually do not show obvious symptoms after CMV infection. Most cCMV-infected infants (approximately 90%) are born healthy, with no significant symptomatic clinical abnormalities, while 10% of infants have clinical symptoms during the newborn period [8]. Symptoms in infants with cCMV range from a single symptom to multiple organ involvement. Mildly symptomatic cCMV disease has been defined as one or two isolated manifestations that are mild and transient (e.g., mild hepatomegaly or isolated petechiae), while moderately to severely symptomatic infection includes multiple manifestations, such as thrombocytopenia, petechiae, hepatomegaly, splenomegaly, and hepatitis [11]. Central nervous system involvement, such as microcephaly, seizures, and/or radiographic abnormalities of the central nervous system, is also considered in moderately/severely symptomatic cCMV infection. Infants with isolated SNHL, but no apparent abnormalities to suggest cCMV disease, are considered asymptomatic [12,13]. More than 50% of children with symptomatic infection have hyperbilirubinemia, elevated liver transaminase, or thrombocytopenia. 

Hearing loss caused by CMV infection was first reported in 1964 [14]. Hearing loss can occur in children who are both symptomatic and asymptomatic at birth. Almost 60% of symptomatic infants with CMV infection will suffer from permanent sequelae, with SNHL being the most common abnormality [15], while 10–15% of asymptomatic infants have hearing loss at birth or experience delayed-onset hearing loss [16]. Delayed-onset hearing loss in children born asymptomatic occurs later than in infants with symptomatic cCMV infection. Hearing loss caused by cCMV infection is very heterogeneous. Patients usually present with unilateral or bilateral, or mild to profound hearing loss. Congenital hearing loss occurs in a proportion of neonates with congenital infection, and the mean age at which these neonates are diagnosed with hearing impairment is 27 to 33 months; another subset of infected newborns develops delayed-onset hearing loss, which can occur months or years after birth [17]. Because the phenotype, severity, and progression of hearing loss caused by cCMV infection are highly variable, many experts agree that children with SSNHL (Sudden Sensorineural Hearing Loss) diagnosed with CMV infection must undergo long-term, frequent, and regular audiological testing. Moreover, neonatal screening for cCMV is not practiced in most countries, and SNHL may not be diagnosed until many years later. cCMV may be the underlying cause of many cases of idiopathic SNHL in children. 

The mechanism of hearing loss caused by cCMV infection remains to be clarified. It was found that the lymphatic system showed obvious inflammatory reaction in the infant specimens, and the virus was observed in the endolymph and perilymph [18]. In the mouse model, the cochlea showed persistent inflammation, and the viral antigen was present in the spiral ganglion and vascular stria of the inner ear, and the infected mice showed a decrease in the density of spiral ganglion neurons [19]. The hearing of immunosuppressed guinea pigs infected with CMV was better than that of the control group [20]. Therefore, cCMV infection-associated hearing loss may be caused by the virus directly damaging the inner ear structure or causing inflammation of the inner ear that leads to damage [21]. The current research mainly describes the following aspects.

#### 2.1.1. Inflammatory Response

**Figure 2:**
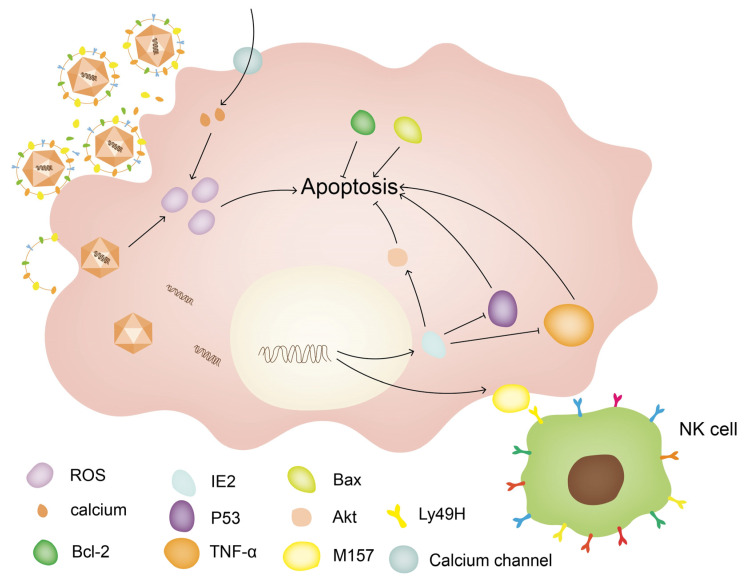
Schematic diagram of the mechanism of CMV-induced cell damage.

CMV infection can induce an inflammatory response and a decrease in Spiral Ganglion Neuron cells (SGN) density, and a persistent inflammatory response can be observed in animal models [22]. Early treatment with methylprednisolone can improve hearing status [19,23,24]. Li et al. speculated that the apoptosis of SGNs may be related to calcium overload or an abnormal ratio of the Bax and Bcl-2 proteins [25]. Another study has shown that CMV can regulate the activity of NK cells for immune evasion, and CMV expresses molecules, such as UL33, US27, US28, and UL78 to bind chemokines to prevent inflammatory cell infiltration [26]. Meanwhile, CMV infection may mediate apoptosis [27] (Figure 2).

#### 2.1.2. Inner Ear Development

cCMV infection could lead to a decrease in Connexin 43 expression. Connexin 43 is associated with the degradation of β-catenin in the Wnt pathway. cCMV infection-induced reduced expression of Connexin 43 may impede the differentiation of sensory progenitors into hair cells and lead to abnormal expansion of the organ of Corti. cCMV infection also could affect the Notch pathway, which may influence number of hair cells and support cells [15]. Studies by Natale et al. suggest that CMV infection may lead to delayed maturation of the auditory pathway, with the opportunity to revert to being normal in the future [28]. 

#### 2.1.3. Inner Ear Homeostasis

After CMV migrates to the inner ear through the blood or cerebrospinal fluid, it primarily infects the stria vascularis. Some studies suggest that hearing impairment may be related to the poor maintenance of inner ear potential caused by striatal dysfunction [29,30,31]. The blood–labyrinth barrier is more permeable in the infected mouse model, and it is speculated that hearing loss may result from changes in inner ear homeostasis caused by cCMV infection, destroying the blood–labyrinth barrier [27].

The diagnosis of cCMV infection includes prenatal diagnosis and neonatal diagnosis. Prenatal diagnosis is based on the serological examination of pregnant women and relies on nucleic acids and antibodies to determine whether there is a primary or chronic CMV infection [32]. Fetal ultrasonography is the primary non-invasive method for evaluating patients with suspected or confirmed cCMV infection [33]. The ultrasound features of common fetal cCMV infection include hyperechoic bowel hydronephrosis, hepatomegaly, periventricular echo density, ventricular dilatation, microcephaly, and global growth retardation [34]. Evidence of active CMV infection includes virus isolation, cytomegalic inclusion bodies, viral antigens, specific viral genes, virus-specific DNA load, and increased anti-CMV IgG titer ≥ four-fold in two sera from the acute phase and the convalescent phase [35]. The diagnosis of cCMV infection requires testing for the virus within the first three weeks of life, as, beyond this period, intrauterine and perinatal infections cannot be distinguished from those acquired after birth [36]. Although screening methods for blood samples showed that the incidence of cCMV in blood was lower than that in urine or saliva [13], virus replication and active infection are mainly identified by serological and molecular methods [37]. Urine samples are subject to waiting and contamination during collection, so their use in screening for neonatal cCMV is limited. A large study showed a low false-positive rate of salivary PCR (0.03% to 0.14%), suggesting that the use of salivary PCR to screen for cCMV infection in breastfed newborns of serologically CMV-positive women remains reliable [38]. The combination of amniocentesis and PCR can assess whether there is a risk of infection and HL in fetuses after maternal infection. For mothers infected with CMV, amniocentesis should be performed at least eight weeks after initial infection and after 18–20 weeks of gestation. 

The TORCH examination has been included among the pre-pregnancy examinations in some countries, including evaluation for cCMV. cCMV hyperimmune globulin (human immunoglobulin [HIG]) has been evaluated as a potential therapy to prevent vertical transmission in women with primary CMV infection during pregnancy. However, the therapeutic efficacy of HIG is still controversial. Recent studies have shown that HIG is ineffective in reducing the risk of CMV in women with primary infection during pregnancy [39]. A prospective, randomized, double-blind, placebo-controlled trial showed a significant decrease in the positive rate of cytomegalovirus amniocentesis after oral administration of valaciclovir in pregnant women with primary cytomegalovirus infection compared to the placebo treatment group [40]. Ganciclovir has shown teratogenic effects in animal studies and, therefore, cannot be used to treat pregnant women with active CMV infection. Currently, there is no licensed vaccine to prevent cCMV infection. The recombinant glycoprotein B vaccine and mRNA CMV vaccine for the prevention of cCMV infection are under development and undergoing clinical trials [41]. We have found evidence of cCMV infection in some children with congenital deafness, but the detection of CMV late after birth cannot determine whether CMV infection is the cause of hearing loss. In countries and regions with a high risk of cCMV infection in newborns, cCMV screening should be performed at birth, in conjunction with the results of neonatal hearing screening [10].

Some researchers have suggested that only newborns with moderate to severe symptoms of cCMV infection should be treated with ganciclovir, and the treatment of CMV-DNA-negative and asymptomatic newborns is not recommended [42]. The efficacy of ganciclovir or valganciclovir as antiviral therapy for cCMV-infected SNHL neonates is also controversial. A series of studies showed that, in enrolled newborns (less than 30 days of age) treated with ganciclovir (6 mg/kg/dose administered intravenously every 12 h for six weeks), five of twenty-four ganciclovir recipients (21%) had worse hearing in their best ear between baseline and ≥1 year compared with 13 of 19 subjects (68%) in the no-treatment group (adjusted *p*-value = 0.002; OR 10.26 [95% CI: 1.79, 81.92]) [43]. In the follow-up study, antiviral therapy was extended from six weeks to six months and conferred a long-term hearing benefit. A controlled placebo trial found that the whole-ear hearing (i.e., the hearing of one or two ears that can be evaluated) of the six-month valganciclovir treatment group was more likely to improve or remain normal at 12 months than that of the six-week valganciclovir treatment group in neonates with symptomatic cCMV disease, and the improvement of whole-ear hearing was maintained at 24 months [44]. A retrospective study found that, after long-term antiviral treatment, infants with isolated SNHL due to cCMV had significantly improved hearing at baseline, and the unaffected ears did not deteriorate (intravenous ganciclovir 5 mg/kg/day for six weeks, followed by oral valganciclovir 17 mg/kg twice a day for 12 weeks and then once a day until the completion of 12 months of treatment; or oral valganciclovir at 17 mg/kg/dose in two daily doses for 12 weeks can be used, and then one daily dose can be used until completion of 12 months). It should be noted that antiviral therapy increases the risk of neutropenia [45]. Compared with the ganciclovir randomized controlled trial, the risk of neutropenia in the first six weeks of valganciclovir treatment is lower. Antiviral therapy must be started within the first month of life.

### 2.2. Herpes Simplex Virus

Herpes simplex virus (HSV) belongs to the family *Herpesviridae*. The incubation period of HSV is two to twelve days. HSV has two serotypes, HSV-1 and HSV-2. HSV-1 transmits mainly through close contact [46], and HSV-2 transmits mainly through sexual contact or via the maternal genital tract to newborns [47]. HSV-1 infections are mainly associated with orofacial herpes, including gingivostomatitis due to primary infection in children, which is often accompanied by fever and blistering injury in the oral cavity, and lip herpes caused by recurrent infection, which manifests as blister clusters at the junction of the oral cavity, lips, and nasal mucosa. Other symptoms include difficulty swallowing, fever, myalgia, and sore throat. Both primary and recurrent infections can cause encephalitis, which can have neurological sequelae. Diseases associated with HSV-2 infection include genital tract herpes, characterized by regional tingling, erythematous papules, and blisters. HSV can remain latent in nerve cells for months to years [47]. The reactivation of viral replication manifests clinically as recurrent ulcerative lesions or sub-clinically as asymptomatic viral shedding. Primary infection causes more severe and long-lasting damage than recurrent infection. The sequelae or complications of primary infection include ocular complications, mucous membrane involvement, encephalitis, hearing loss, mental retardation, microcephaly, systemic diseases, such as gingivitis, esophagitis, and vulvovaginitis, high fever, convulsions, and even death [48]. In 2016, it was estimated that 2/3 people aged 0–49 would have HSV-1 infection, while 13.2% of people aged 15–49 would be infected with HSV-2 [49]. 

In-utero infection and HSV culture-positive postpartum infection remain risk indicators for permanent congenital, delayed, or progressive hearing loss in childhood, and such children should have a routine early hearing test at the age of 24–30 months [50]. The reported incidence of SNHL in case series of neonates infected with HSV ranges from 0% to 33% [51]. Hearing loss caused by HSV infection in newborns can be bilateral or unilateral, mild to severe SNHL, with or without severe neurological complications. Kaga et al. described four cases of children with encephalitis caused by HSV infection, whose hearing loss ranged from mild to moderate. Computed tomography (CT) and MRI scans of their brains showed common bilateral lesions in the auditory cortex [52]. The reactivation of latent HSV-1 infection after infancy can also cause hearing loss [53]. Rabinstein et al. described a 61-year-old woman with severe bilateral hearing loss who experienced serological reactivation of a previously latent HSV-1 virus. MRI performed after the onset of deafness showed bilateral enhancement of the seventh and eighth nerve complexes in the ventricle, consistent with inflammatory neuritis. She was treated with acyclovir and methylprednisolone, and her hearing did not return [54]. 

HSV antigen and virus capsid could be detected in the cochlea of virus-infected animal models, while tympanum, vestibular fibrosis, outer hair cell loss, and vascular atrophy were observed in the HSV-infected cochlea [55]. Morphological changes, such as atrophy, curling, and spot-like formation were observed in the tunica of guinea pigs inoculated with HSV on one side of the tympanum, and HSV antigen could be detected in the uninoculated side of the cochlea [55]. The pathology of viral labyrinthitis in temporal bone samples of patients with sudden deafness is consistent with that observed in animal experiments [56]. Stokroos et al. found earlier hearing recovery and less cochlear damage in guinea pigs infected with HSV-1 treated with a combination of prednisone and acyclovir, suggesting that hearing loss caused by HSV-1 infection may be related to viral inflammation [57]. Because the phenotype of virus-induced hearing loss is very similar to that of SSNHL, some researchers have conducted serological tests for various deafness-related viruses in patients with hearing loss, and the results showed that a considerable portion of people had HSV antibodies in their serum [58,59,60,61]. 

All pregnant women with primary HSV infection should be offered antiviral therapy regardless of timing of occurrence during pregnancy [62]. Suppressive antiviral therapy is recommended at 36-week gestation through delivery. Cesarean delivery is recommended if the mother was diagnosed with primary genital herpes within the six weeks before delivery [63]. For patients suspected of having primary meningitis due to HSV infection, HSV DNA in the CSF can be detected by PCR to confirm the diagnosis [64]. Treatment for hearing loss associated with HSV infection includes antiviral and steroid therapy. Combination treatment, consisting of prednisolone and acyclovir, may result in earlier hearing recovery and less extensive cochlear destruction compared to the use of prednisone or acyclovir alone [57]. Hearing loss patients who have not recovered after treatment with antiviral drugs and steroids should receive treatment with hearing aids or cochlear implants.

### 2.3. Varicella-Zoster Virus

Varicella-zoster virus (VZV) is a pathogen that causes chickenpox and herpes zoster. VZV, HSV-1, and HSV-2 belong to the alpha subfamily of herpesviruses, thus VZV is also known as human herpesvirus type 3. The host range of VZV is highly limited to humans. In a systematic review of research conducted from 2002 to 2018, researchers estimated its cumulative incidence to be about 2.9–19.5 cases per 1000 people, most of whom were women [65].

VZV can spread via droplets or directly through contact with fluid in vesicles, enter the body through the respiratory tract, spread rapidly from the pharyngeal lymphatic tissue to circulating T lymphocytes, and cause viremia [66]. After 10–21 days, VZV reaches the skin, producing multiple umbilicated and painful vesicles [67]. After primary infection, VZV enters a latent state, mainly existing in the neurons of peripheral autonomic ganglia and running through the entire nerve axis, including cranial nerve ganglia, such as dorsal root ganglia and trigeminal ganglia, and autonomic ganglia in the enteric nervous system [68]. After decades, latent VZV can be reactivated either spontaneously or under the influence of multiple factors. Activated VZV is transported within the sensory axons and infects epithelial cells, thus producing a characteristic rash in the skin area innervated by a single sensory nerve [69]. Risk factors for viral reactivation include age > 50 years, immunosuppression, diabetes, gender, genetic predisposition, physical trauma, and recent psychological stress [70]. VZV could be transported across the placental barrier, so infection in pregnant women may lead to intrauterine fetal infection; fortunately, vaccination can confer specific protection to the fetus [71]. 

Reactivation of VZV has been reported as a cause of SSNHL [72,73,74]. Latent VZV reactivation causing inflammation of geniculate ganglions and facial nerve may lead to Ramsay Hunt syndrome (RHS) or ocular herpes zoster (herpes zoster ophthalmicus [HZO]). Symptoms in patients with RHS include ipsilateral peripheral facial paralysis, ear herpes, and ear pain, possibly accompanied by SNHL, tinnitus, and vertigo [75]. The chickenpox zoster virus that lurks in the ophthalmic branch of the trigeminal nerve may cause HZO, and patients may exhibit eyelid edema, eyelid ptosis, keratoconjunctivitis, acute uveitis, outer sclera, or scleritis. The incidence of hearing loss in VZV infection cases reported in the literature varies from 7% to 85% [76]. Although hearing thresholds are affected at all frequencies, VZV-induced hearing loss is mainly concentrated at high frequencies [76,77,78]. Kim et al. found that patients with vertigo had a greater difference in hearing between the two sides, while patients with facial paralysis had no significant difference in bilateral hearing loss [77].

Histopathological studies have shown inflammatory cell infiltration in the vestibular and cochlear ganglia and degeneration of the inner ear end organs in patients with RHS [79,80]. Although the mode of transmission of VZV infection between nerves and to the end organs of the inner ear is unclear, transmission through the round and oval windows has been suggested as a possible route of inner ear involvement [81]. According to current studies, patients’ hearing loss may be caused by cochlear and/or post-cochlear factors [82,83,84], while vestibular dysfunction may be due to inflammation of the vestibular nerve [85,86] or lesions of the end organs [87], or even both [88].

The diagnosis of RHS and HZO usually depends on history, physical examination, and a serological test or PCR for VZV in the blood or cerebrospinal fluid. The standard therapy for VZV is acyclovir or valganciclovir [89]. In some patients exhibiting acyclovir resistance, famciclovir is an alternative. Valganciclovir is currently used to treat infections of CMV, which is in the same family as VZV, and the first successful treatment of varicella-like disease using valganciclovir was reported in 2014 [90]. In addition, a helicase-primase inhibitor, called amenamevir, which inhibit the formation of replication forks, is also used to treat VZV and is currently approved in several countries [91].

For the prevention of herpes zoster, two vaccines are currently available. One is a live-attenuated VZV vaccine called Varivax^®^ (Vaccine Oka, vOka) (Merck Sharp & Dohme, Rahway, USA), based on the VZV attenuated Oka strain, and the other is a recombinant, adjuvanted VZV glycoprotein E subunit vaccine called Shingrix^®^ (GlaxoSmithKline plc, London, UK). Shingrix^®^ is not approved for the prevention of chickenpox in children. A VZV immunoglobulin preparation, VariZIG^®^ (Kamada, Rehovot, Israel), can be used for prophylaxis in groups at high risk of severe disease that lack immunity to VZV and are not suited to receive the VZV vaccine.

### 2.4. Epstein-Barr Virus

Epstein-Barr virus (EBV) belongs to the herpesvirus family of viruses. EBV contains double-stranded linear DNA of 170–175 kb, which is transmitted mainly via the salivary transfer of EBV-infected B cells and by aerosol [92]. EBV is widespread in the population, with more than 90% showing seropositivity [93]. 

EBV is usually spread through contact with respiratory secretions [94]. The symptoms of EBV infection vary greatly depending on the patient’s age and immune status. Most younger children have no apparent symptoms at the first infection [95], while a few develop pharyngitis and upper respiratory tract infections. Thirty to fifty percent of cases manifest clinically as infectious mononucleosis (IM) if occurring in adolescents or adults. About half of the patients with primary EBV infection present with IM [96]. Patients with low immune function may show typical symptoms and complications of EBV infection [93]. 

EBV detection in SSNHL cases has been reported sporadically over the years. Williams et al. reported that evidence of altered cell-mediated responses to Epstein-Barr virus antigens have been found in three young patients with sudden permanent hearing loss one to four months before the onset of hearing loss [97]. Erzurum et al. reported a 16-year-old boy with symptoms and signs of cerebellar ataxia and hearing loss. Results of a test for infectious mononucleosis showed positive and a fourfold rise of serum antibody titer to Epstein-Barr virus capsid antigen [98]. Yossepowitch assessed 14 SSNHL patients infected with EBV and summarized that sensorineural hearing loss usually occurred in young people with IM, with women accounting for a large proportion of cases (M:F = 5:9). SNHL usually occurs in the recovery period of the disease, but symptoms may be rare. The average time from acute disease to hearing loss was six weeks, ranging from 2.5 weeks to 3.5 months [99]. The total percentage of patients with bilateral SNHL was 4–17% [99]. However, of 14 (43%) cases of SNHL caused by EBV infection, six involved bilateral ears. Hearing loss is mainly bilateral, and mild to severe damage mainly occurs at middle and high frequencies [99]. Only 21.4% of patients out of 14 cases recovered completely, and 21.4% recovered slightly, while the rest developed permanent hearing loss. In cases without tinnitus, the milder and lower the frequency of damage, the greater the likelihood of recovery.

Buza et al. reported a case of a young man diagnosed with EBV-associated hearing loss for four years [100]. Wong et al. reported on a 42-year-old patient with total-frequency severe deafness in the right ear and tinnitus in the right ear; the plasma EBV DNA level of the patient as detected by PCR increased to 4.1 × 10^4^ IU/mL (reference range: undetectable) [101]. The patient was finally diagnosed with EBV + NK cell leukemia in the central nervous system, and a unilateral putamen lesion resulted in contralateral deafness [101]. In 2019, Li et al. reported a case of a child with progressive hearing loss at one month of EBV infection and complete loss at forty days; ear and neurological examinations revealed no organic lesions, and it was hypothesized that the cause of deafness in the child could be damage to the functional areas of the cortical layer of the central nervous system triggered by EBV infection [102]. Williams et al. suggested that, in some susceptible individuals, EBV infection can reactivate the virus in the inner ear, causing pathological damage [97]. Another study examined the histopathology of the temporal bone in patients with sensorineural deafness, revealing atrophy of the cochlear capsule, stria vascularis, cochlear nerve, and vestibular organs. The researchers suggested that viral particles may reach the cochlea through the bloodstream to replicate locally and undergo rapid pathophysiological changes, resulting in reduced blood flow to the inner ear, which is reversible to some extent, but may also cause greater damage, leading to permanent hearing loss [103]. In addition, Arslan et al. reported on a 43-year-old male with a history of EBV infection who developed sudden hearing loss in both ears [96].

## 3. Severe Acute Respiratory Syndrome Coronavirus 2

Severe acute respiratory syndrome coronavirus 2 (SARS-CoV-2) belongs to the family of *Coronaviridae* and is an enveloped virus containing a single-stranded, positive-sense RNA genome (+ssRNA) [104]. SARS-CoV-2 has a zoonotic origin, and its hosts include humans, cats, bats, civets, dogs, and camels, among other vertebrates [105]. The main transmission routes of SARS-CoV-2 include droplet, contact, aerosol, and fecal–oral transmission.

The common initial symptoms of SARS-CoV-2 infection include fever, cough, sore throat, headache, muscle pain, diarrhea, and difficulty breathing. It mainly affects the human respiratory system, but it can also harm the central nervous, cardiovascular, gastrointestinal, hepatobiliary, and renal systems [106]. In rare cases, SARS-CoV-2 infection affects the peripheral nervous system and muscles, resulting in Guillain-Barré syndrome, Miller-Fisher syndrome, polyneuritis cranialis, and rhabdomyolysis. In addition, there are extensive studies on the effects of infection with SARS-CoV-2 on hearing, smell, and vertigo [107,108,109,110,111,112,113]. However, doctors often only focus on the classical symptoms of SARS-CoV-2 patients, ignoring their hearing loss, tinnitus, dizziness, vertigo, anomalous sense of smell and taste, and other symptoms. 

Much of the literature has reported the relationship between SARS-CoV-2 and sudden sensorineural hearing loss (SSNHL); it spans the period from 2020 to 2022, is not limited to a particular country or region, and suggests that many SARS-CoV-2 variants can cause otological symptoms. Ong et al. reported that 68% of patients presenting with otological symptoms began to experience hearing loss within one month of the onset of the typical symptoms or diagnosis of SARS-CoV-2 infection, and 23.1% presented with SSNHL [114]. Of those for whom recovery data was available, approximately 56% recovered entirely or were able to reach baseline hearing thresholds, with 90% recovering within two weeks and the rest within one month. Tinnitus, vertigo, ear pain, and a sense of fullness in the ear may also be complications of SSNHL and even the primary symptoms for which patients seek medical attention [114]. Dusan et al. also reported that 30 (40.5%) SARS-CoV-2-positive patients had sensorineural hearing loss (SNHL), and there was a statistically significant difference in its incidence rate between novel coronavirus-positive patients and the control group [115]. Meng et al. reported that 60.9% of SSNHL patients associated with SARS-CoV-2 had tinnitus symptoms similar to those of conventional SSNHL [116]. Statistically, it was found that most patients with SARS-CoV-2 infection present with unilateral SNHL, and a small number of patients may develop bilateral hearing loss, mostly at high frequencies, with the severity ranging from mild to complete deafness [117,118]. Researchers have detected different variants of SARS-CoV-2 worldwide at different times and found that their genomes are undergoing profound changes. Significant differences exist between different variants regarding transmissibility, pathogenicity, mortality, and symptoms. The virus cannot be guaranteed to constantly mutate in the direction of weakened virulence. It is worth noting that chloroquine and hydroxychloroquine are used to treat SARS-CoV-2 infection in some countries [119]. These drugs have ototoxicity, and the dose used for SARS-CoV-2 infection treatment is much higher than that used for malaria treatment. Therefore, some hearing loss may be caused by the ototoxicity of drugs.

The mechanism of hearing loss caused by SARS-CoV-2 is still not clear. It has been reported that SARS-CoV-2 may cause hearing loss by damaging the auditory center of the brain and cochlea. The spike protein of SARS-CoV-2 binds to its host cell receptor angiotensin-converting enzyme 2 (ACE2) and is hydrolyzed and cleaved by the transmembrane protease serine 2 (TMPRSS2) protein, which pulls the virus and host membrane together to initiate the fusion of the virus and cell membrane and facilitate viral entry [120]. The amplified virus leaves the host cell and binds to the abundant ACE2 receptors in the auditory center of the brain, further leading to hearing loss [106]. SARS-CoV-2 may also enter the brain upstream through the olfactory fissure and disrupt the blood–brain barrier [121]. SARS-CoV-2 can affect red blood cells and cause their deoxygenation. Thus, SARS-CoV-2 may lead to continuous hypoxia, which also could do damage to the auditory center of the brain [122]. SARS-CoV-2 was found to colonize the middle ear and mastoid in gross autopsies of COVID-19 [123]. Jacob et al. reported a case of a patient who presented with systemic symptoms and developed hearing loss with total deafness after brief treatment, followed by rapid recovery. Supportive care resolved her hearing loss [124]. One patient was found to have a vagal hemorrhage via magnetic resonance imaging (MRI) [108]. Mustafa et al. performed transient evoked otoacoustic emission (TEOAE) in 20 asymptomatic infected individuals. Despite the absence of symptoms, high-frequency pure-tone thresholds and TEOAE amplitude of the asymptomatic infected individuals worsened, suggesting that SARS-CoV-2 infection may have deleterious effects on hair cell function [125]. Using human-induced pluripotent stem cell (hiPSC)-derived in vitro models, Jeong et al. demonstrated that inner ear tissues co-express ACE2 and other proteins required for virus entry and that the virus may cause direct damage to the inner ear [126]. In addition, elevated D-dimer and microthrombosis were detected in the blood of patients with SARS-CoV-2 infection. Microthrombi may also be involved in the development of hearing loss [127]. 

Kandimalla et al. have summarized various vaccines’ epidemiology, effectiveness, and status, which we will not repeat here [128]. SARS-CoV-2 infection is very contagious and pathogenic; thus, the risk of adverse reactions or reinfection from these vaccines has not been well regarded. Many otolaryngologists and medical workers are concerned that vaccination can cause ear symptoms. Wichova et al. reported that some SARS-CoV-2 infection patients developed new or significantly aggravated ear symptoms, including hearing loss, tinnitus, dizziness, and vertigo, shortly after receiving the Pfizer vaccine [129]. Formeister et al. evaluated the clinical features of 86,553,330 SARS-CoV-2 vaccinators in the United States (U.S.) between 14 December 2020 and 2 March 2021, and they found that the incidence of SARS-CoV-2-related SSNHL after vaccination was not higher than that in the general population [130], whereas Junhui Jeong reported three patients who developed SSNHL within three days of SARS-CoV-2 vaccination [107]. However, more evidence is needed to determine the relationship between different vaccines and ear symptoms, such as hearing loss [131]. Zoccali et al. recently reported two cases of SSNHL after a third dose of the SARS-CoV-2 mRNA vaccine. Notably, one of these patients had never previously tested positive for SARS-CoV-2 [132]. Hypotheses regarding SARS-CoV-2 vaccine-induced SSNHL include viral reactivation, reduced clinical duration of SARS-CoV-2, known potential risk of ear symptoms, and migraine exacerbations [129,133,134,135,136]. More evidence is needed on the association between SARS-CoV-2 vaccination and the increased risk of SSNHL. However, post-vaccination SSNHL is still of concern and requires systemic or intratympanic steroid administration [107].

Studies have shown a risk of vertical transmission of SARS-CoV-2 [137]. The ACE2 receptor is significantly expressed in the placenta; therefore, SARS-CoV-2 may bind to the ACE2 receptor and enter the fetus [138]. Nevertheless, SARS-CoV-2 isolates were not found in the amniotic fluid or breast milk samples of infected pregnant women, nor were they found in the neonatal throat swabs and cord blood of newborns [138,139,140,141,142]. On the other hand, a few SARS-CoV-2-positive pregnant women gave birth to infected newborns, so whether SARS-CoV-2 can transmit vertically and cause neonatal hearing loss is controversial [143,144,145]. One (out of 251, 0.4%) baby born to a mother who tested positive in the third trimester of pregnancy was positive for SARS-CoV-2 in a report published by the World Association of Perinatal Medicine (WAPM), indicating a low risk of vertical transmission [146]. In another report, of 205 babies born to SARS-CoV-2-positive mothers, 6.3% (13/205) tested positive for SARS-CoV-2 [144]. Anti-SARS-CoV-2 antibodies have also been detected in neonates that have tested negative for SARS-CoV-2, so the risk of vertical transmission of SARS-CoV-2 cannot be completely ignored. Multiple studies have shown that SARS-CoV-2 infection in pregnant women does not cause congenital hearing loss in their newborns [137,145,147]. In contrast, Mehmet Akif Alan showed that a positive PCR for SARS-CoV-2 during pregnancy was significantly associated with an increased risk of infection with abnormal newborn hearing screening (NHS) outcomes [148]. More evidence must be gathered to determine the relationship between infection during pregnancy and the risk of neonatal hearing loss. 

SARS-CoV-2 is still prevalent worldwide, and there is no specific treatment for SARS-CoV-2 -related hearing loss. Systemic supportive therapy and oral, intravenous, and intra-drum steroid injections play a role to some extent, but some patients still fail to experience significant efficacy [127].

## 4. Hepatitis B Virus

Infection with hepatitis B virus (HBV), which belongs to the family *Hepadnaviridae* and genus *Orthohepadnavirus*, is a global public health concern. The number of HBV carriers in the world is estimated to be as high as 370 million. China is a country with a high endemicity of hepatitis B, and the overall population HBV carrier rate is about 7.18% [149,150]. The main transmission routes of HBV are blood, blood products, and iatrogenic transmission, vertical transmission, sexual transmission, and close contact transmission. The clinical manifestations of HBV infection are diverse, including asymptomatic HBV carriers, acute hepatitis, chronic hepatitis, and severe hepatitis, as well as serious diseases, such as liver cirrhosis and liver cancer [151]. 

The number of SNHL in HBV-infected patients is approximately 0.92 per 10,000 people per year [152]. Nasab et al. reported that pure tone average (mean thresholds of 500, 1000, and 2000 Hz) for left and right ears of HBV-infected patients are statistically significantly higher than the control group, which show that HBV-infected patients are more likely to suffer from hearing loss [153]. Bao et al. reported that the average hearing thresholds (HTs) of a control group were 10.70, while the HTs of HBV infected group were 12.42 dBHL. The frequency-specific HTs of 114 ears in the control group and HBV infected group were statistically different for hearing frequencies, ranging from 250 to 8000 [154]. Parizad et al. reported significant differences in average pure tone audiometry (PTA) and hearing loss between 83 HBsAg-positive patients and 108 HBsAg-negative patients in a case-control study. There were also significant differences in mean PTA between the two groups at 250, 4000, and 8000 Hz speech frequencies [155]. 

Similarly, Tsai et al. selected 279 patients from an HBV cohort and 845 patients from a control cohort without HBV infection in Taiwan to investigate the risk of developing SSNHL in patients with HBV. The study showed that the incidence of SSNHL was 1.33 times higher in the HBV group than in the control group [152]. Islam et al. investigated the association of HBV infection with hearing impairment using a representative sample of the Korean population. Among 6583 men and 8702 women (older than 20 years) enrolled in the survey, subjects who were HBsAg-positive had a lower average of pure-tone thresholds and lower prevalence of hearing impairment at both low/mid and high frequencies compared to those who tested negative, and a significant negative association between HBV infection and high-frequency mild hearing impairment remained [156]. This result is contrary to what was reported in previous studies. The results of retrospective studies conducted in the population are somewhat controversial, possibly due to insufficient adjustment for confounding factors in different studies. Further research is needed to determine whether HBV infection can cause hearing loss. Notably, Sood et al. reported a case of Vogt-Koyanagi-Harada Disease after the administration of a hepatitis B vaccine with clinical symptoms of hearing loss and tinnitus [157].

Polyarteritis nodosa caused by HBV infection may be the cause of hearing loss in HBV patients [158]. However, no polyarteritis nodosa was found in a clinical survey of 95 HBV-infected patients conducted by Nasab et al. [153]. Feng et al. speculated that hearing loss might be caused by HBV infection of the auditory nervous system or the deposition of immune complexes in the internal auditory artery, resulting in partial or complete occlusion leading to insufficient blood supply [159]. Abnormal inner ear immune system and viral load may also be the cause of HBV induced hearing loss [154,160].

## 5. Human Immunodeficiency Virus

Human immunodeficiency virus (HIV) is a lentivirus belonging to the subfamily *Orthoretrovirinae* of the family *Retroviridae*. 650,000 people died of acquired immune deficiency syndrome (AIDS) in 2021, with about 1.5 million new HIV infections [161]. The main modes of transmission include sexual transmission, blood transmission, and vertical transmission.

There is a significant difference in the prevalence of hearing loss between HIV-infected and HIV-negative people. About 13.1–38.8% of HIV-positive children have some kind of hearing loss [162,163,164], including congenital or postlingual, progressive, or sudden hearing loss [165]. A cross-sectional study by Hrapcak et al. found that 24% of HIV-infected children had hearing loss, mainly conductive hearing loss, which accounted for about 82%, and SNHL, which accounted for 14% of cases [166]. Although unilateral and bilateral hearing loss have been reported, some researchers believe that HIV infection often causes an opportunistic infection that leads to otitis media, so the hearing loss should be unilateral [163]. The PTA results of HIV-infected people were significantly higher than those of ordinary people in both high-frequency and low-frequency ranges [167]. Mild and moderate hearing loss often occurs in HIV-infected individuals [164,167,168]. The study found that HIV-infected people are more likely to have an abnormal tympanogram, and after adjusting for confounding factors, their distortion products otoacoustic emissions (DPOAE) results are also lower [169]. 

Hearing loss related to HIV infection may result from direct damage by the virus or auditory pathway damage caused by inflammation; ototoxic drugs may also be related to hearing loss [165]. Jr et al. detected virus particles with HIV morphological characteristics in the stria vascularis and tegmental area [170], indicating that the virus may enter the inner ear. Maro et al. showed lower distortion product otoacoustic emissions (DPOAEs) in HIV infection individuals compared with controls, which suggests the direct impact of HIV infection on the auditory efferent pathway or cochlea [169]. HIV infection exhibits characteristic brain abnormalities, such as cortical atrophy and decreased white matter density, which may affect the central nervous system responsible for speech development and lead to impaired language ability in patients [171]. HIV also attacks the human immune system, increasing opportunistic infection and leading to conductive hearing loss [172]. In addition, the infection rate of CMV in infants exposed to HIV is relatively high [173]. At present, the early start of antiretroviral therapy, reduction in mitochondrial DNA content induced by nucleoside analog reverse transcriptase inhibitors, and mitochondrial DNA mutations associated with aging and HIV-1 infection may lead to hearing impairment in older patients with HIV-1 infection [174], that is to say, many factors may cause hearing loss. 

HIV infection-induced conductive hearing loss can be corrected with hearing aids, but infectious complications and low immunity may limit their application. For patients with HIV infection related SNHL, after evaluating their surgical tolerance, hearing can be improved with cochlear implantation [165]. Although active antiviral therapy may lead to drug-induced hearing loss and developmental defects persist in HIV patients compared with healthy children, it is recommended that ART be used as soon as possible to alleviate the progression of stunting [175].

## 6. Rubella Virus

Rubella virus is a member of genus *Rubivirus*, family *Togaviridae*, transmitted via respiratory droplets and direct contact. Children’s rubella mainly manifests as fever and a mild measles-like rash, with retroauricular and suboccipital lymph node swelling, while infection symptoms are more severe in adults. In addition to the rash, arthritis and joint pain, thrombocytopenia, and encephalitis occur after the rash. Rubella virus infection causes the most harm through vertical transmission. In the first 12 weeks of pregnancy, the risk of congenital infection and congenital disabilities is the highest [176], causing miscarriage, fetal death, fetal malformation, and other congenital rubella syndromes [177]. Before the vaccine’s introduction, up to four infants per 1000 live births were born with congenital rubella syndrome [178]; such children may develop hearing impairment, eye and heart defects, and other lifetime disabilities, including autism, diabetes, and thyroid dysfunction.

The literature shows that 21% of hearing loss among children in Brazil is from the vertical transmission of rubella virus, and the incidence of deafness in children whose mothers have rubella during pregnancy is close to 30%, manifested as bilateral profound hearing loss in most cases [179]. In addition to congenital hearing loss, delayed deafness has been reported in the first year of life [180]. Such hearing loss may be caused by the direct damage by the virus, since in pathological examinations, sporadic cell damage was found in the inner ear, mainly in the cochlear duct and stria vascularis [181,182], and damage to the stria vascularis may lead to endolymphatic changes in composition [4].

There is currently no specific antiviral therapy for rubella, but it can be prevented by vaccination, which is vital given the high incidence of deafness among the children of rubella-infected mothers during pregnancy. By the end of 2020, the rubella vaccine had been introduced in 173 WHO member states, with an estimated global coverage of 70% [183].

It is recommended that infant hearing screening be performed, preferably in the first month of life, if mothers acquire a primary infection during pregnancy. Treatment of hearing impairment includes the use of hearing aids or cochlear implants [184].

## 7. Zika Virus

Zika virus (ZIKV) is an arthropod-borne arbovirus belonging to the *Flaviviridae* family. ZIKV is mainly transmitted through the bite of infected mosquitoes, with *Aedes aegypti* and *Aedes albopictus* being the main vectors [185,186]. The clinical phenotypes of congenital ZIKV infection include brain calcification, microcephaly, intrauterine growth restriction, and fetal death. CT and MRI of the brains of neonates with congenital infection in Brazil revealed hypoplasia of the cerebellum and brainstem, ventricular hypertrophy, delayed myelination, enlarged cisterns, abnormal corpus callosum, calcifications, and cortical malformations [187]. ZIKV is relatively less neuroinvasive in adults, rarely causes meningitis and encephalitis [188], and is associated with an autoimmune disease, Guillain-Barre syndrome, although at a low rate [189]. 

Much evidence of the link between ZIKV and hearing loss emerged during the ZIKV outbreak in Brazil that began in May 2015. Leal et al. assessed hearing in 70 children with microcephaly following ZIKV infection, 5.8% of whom developed SNHL (excluding one child using the ototoxic drug kanamycin), ranging from mild to severe and being unilateral or bilateral. SNHL occurs predominantly in infants whose mothers developed a rash in the first trimester of pregnancy, with all infants with profound hearing loss having severe microcephaly [190]. Lage et al. counted 102 children diagnosed with microcephaly at birth who had gestational exposure to ZIKV. Among the mothers of 102 children with microcephaly, most mothers (81%) reported symptoms of ZIKV infection during the first trimester, especially skin rashes. The sensorineural screening suggested hearing loss in 17.3% and visual impairment in 14.1% of the infants [191]. Almeida et al. conducted a cross-sectional study of eighty-eight one-year-old children with normal hearing who were diagnosed with congenital Zika syndrome, of whom 44.3% demonstrated a delay in hearing acuity [192]. Animal studies have shown that ZIKV can be detected in all areas of the inner ear, associated ganglia, and the surrounding periosteum interstitium following injection of ZIKV into the ear capsule/cup on days two to five of chick embryos, with a significant increase in nerve cell death. This pathological phenomenon may be related to the activation of macrophages [193]. 

The gold-standard method for diagnosing ZIKV infection is the real-time polymerase chain reaction (RT-PCR) test of serum, saliva, or urine during the first few days of acute infection [194]. After the acute phase, serological testing can be performed; however, due to cross-reactivity between ZIKV and other viruses such as the dengue virus and chikungunya virus, further testing is necessary to confirm the presence of neutralizing antibodies [195].

## 8. West Nile Virus

West Nile virus (WNV) is a member of the genus *Flavivirus*. WNV is rarely transmitted from person to person. It spreads mainly through the bite of infected mosquitoes, and a minimal number of people are infected through organ transplants, blood transfusions, and breast milk [196]. It has also been reported to be transmitted through the placenta [197]. About 20% of people infected with WNV develop West Nile fever, whose symptoms include fever, headache, tiredness, general soreness, nausea, and vomiting, occasionally accompanied by a rash and enlarged lymph glands, and the remaining 80% are asymptomatic. It is estimated that one in 150 people infected with WNV develop severe diseases and may develop neuroinfectious diseases, such as West Nile encephalitis, meningitis, or West Nile polio [198].

Hearing loss is very rare in patients infected with West Nile virus. The first case of WNV infection with deafness was reported in 2006 by McBride et al. A 57-year-old white woman with WNV infection was diagnosed with SNHL of moderate severity. During hospitalization, the patient received medications, such as piperacillin/tazobactam, vancomycin, prednisone, azathioprine, and acetaminophen, as well as supportive treatments, such as intravenous fluids, nutritional support, and incentive spirometry, which resulted in hearing improvement [199]. Casetta et al. reported a case of bilateral asymmetric HL in a patient with WNV infection [200]. Szatmary reported on a 57-year-old African American patient diagnosed with acute WNV infection who exhibited bilateral sensorineural deafness and hearing loss that did not improve at discharge (40 days) [201]. Weatherhead examined short-term (one to three years) and long-term (eight to eleven years) neurological status in 60 patients infected with WNV in the Houston West Nile Cohort (HWNC). The short-term neurological status of 60 WNV-infected patients showed hearing loss in twenty, gait instability in four, and tremor in eight [202]. Parrino et al. reported on two patients with WNV infection and hearing loss [203]. A 56-year-old man was confirmed as having WNV infection and complained of bilateral hearing loss and tinnitus; tympanum measurements showed an A-type curve. Prednisone at 1 mg/kg for 15 days with tapering therapy resulted in no improvement in hearing at five and twenty-five days and improved hearing at seven months. Another 75-year-old male patient diagnosed with WNV infection developed progressive bilateral SNHL on day five of hospitalization, with left and right PTAs of 55 and 53.7 dB, respectively, compared with a right ear PTA of 22.5 dB and left ear PTA of 36.2 dB before infection. He was also diagnosed with right horizontal canal positional vertigo (apogeotropic variant). At follow-up seven months later, the patient reported that his hearing and balance gradually returned to normal. In addition, Jamison et al. reported a case of a WNV patient with HIV infection and hearing loss [204].

WNV can be tested in different ways, such as with ELISA for antibody testing, RT-PCR for virus detection, or cell culture for virus isolation. For symptomatic patients, the primary treatment is symptomatic supportive treatment [205].

## 9. Human Enterovirus

Human enteroviruses (EVs) were originally classified according to their pathogenicity. *Enterovirus* belongs to the picornavirus family and is divided into 12 types: enterovirus A to H, enterovirus J, and rhinovirus A to C. There are many serotypes of EVs due to the mutation of viral structural proteins. The serotypes that cause human diseases are formally divided into five categories: poliovirus, coxsackievirus, rhinovirus, enterovirus, and echovirus [206]. EVs may cause fever, conjunctivitis, hand, foot, and mouth disease, and other serious diseases, such as pneumonia, meningitis, myocarditis, pericarditis, encephalitis, and paralysis [206]. 

Schattner et al. reported on a 27-year-old male patient with sudden severe bilateral hearing loss and tinnitus. PTA showed that the right ear’s hearing threshold was 60 dB, that of the left ear 65 dB, and the discrimination score was 76%. All measuring frequencies from 250 to 8000 Hz were affected. Brain imaging (CT contrast and MRI gadolinium), cerebrospinal fluid biochemistry, and cytology were normal. The patient received methylprednisolone pulse therapy (1 g/day intravenous injection lasting for three days) and then used prednisone (60 mg/day, gradually decreasing). After five days, the patient’s hearing improved, with speech reception thresholds of 15 dB (right ear) and 25 dB (left ear) at all measured frequencies and 100% discrimination. EV (echovirus type 4) was subsequently cultivated from the stool of the patient [207]. Thus, steroid hormones may be helpful for hearing loss caused by some types of enterovirus infection. Tekin et al. described a patient suffering from multiple progressive diseases, including accidental weight loss, fever, fatigue, myopathy, pancreatitis, and sensorineural hearing loss. Coxsackie virus A9 was detected in the patient’s skin, and enterovirus RNA was also detected in the cerebrospinal fluid and muscle [208]. In addition, many studies detected EVs in a considerable number of SSNHL patients via PCR. Mentel et al. analyzed for the presence of enterovirus RNA by RT-PCR on 48 unselected patients with unilateral idiopathic SSNHL, which showed one patient (2.08%) with enterovirus infection [209]. Gross et al. also detected one enterovirus RNA-positive plasma sample from a group of 48 patients with idiopathic SSNHL [210]. Kadambari et al. found that the incidence rate of infant EV meningitis is higher, and meningitis is a common cause of hearing loss, which may be one of the mechanisms of hearing loss caused by EV [211]. A retrospective study analyzed 1028 enterovirus culture-positive cases from January 1995 to June 2003, including three hundred thirty-three cases involving the central nervous system, two hundred eighty-two cases of aseptic meningitis (84.7%), forty-four cases of encephalitis (13.2%), seven cases of encephalomyelitis/poliomyelitis-like syndrome (2.1%), and one case of hearing loss [212].

Some studies have shown the ability of various gut viruses to invade the cochlea and their correlation with hearing loss. A complete absence of the cochlear nerves and substantially reduced peripheral and central axes with loss of some inner hair cells was observed in a study carried out on temporal bone specimens of 26-month-old white female with a paralytic syndrome clinically and pathologically identical to poliomyelitis [213]. The coxsackievirus and adenovirus receptor (CAR) is an essential regulator of cell growth and adhesion during development [214]. In newborn mice, CAR is located at the junction of most cochlear cell types, while in adult mice, CAR is located in Sertoli cells and stria cells. The ability and targeting of Coxsackie virus B and other viruses to enter inner ear cells may be related to the distribution of cochlear CAR [215].

## 10. Lassa Virus

Lassa virus (LASV) is the causative agent of Lassa fever, a common disease in West Africa [216]. The natural host of LASV is *Mastomys natalensis* rodent [217]. Its transmission may occur via humans ingesting infected animal excrement, eating contaminated food or infected animals, exposing open wounds to virus-contaminated environments, or inhaling polluted air. 

The incubation period for Lassa fever is 2 to 21 days. About 80% of people infected with LASV are asymptomatic, and mild symptoms can be relieved within a week [218]. In terms of symptoms, the onset of the disease is usually gradual, with fever, general weakness, and discomfort initially. Headache, sore throat, muscle pain, chest pain, nausea, vomiting, diarrhea, cough, and abdominal pain may occur after a few days. In severe cases, swelling of the face, pleural effusion, bleeding from the mouth, nose, vagina, or gastrointestinal tract, and low blood pressure can develop. Proteinuria may also occur. Shock, seizures, tremors, disorientation, and coma are elements of late-stage infection [219].

In a study by Mateer et al., an average of 33.2% of Lassa fever survivors developed unilateral or bilateral SNHL, and these SNHL cases were identified at 10 to 15 days after the onset of Lassa fever symptoms or at the convalescent stage of the disease [219]. In a case–control study by Cummins et al., 29% of LASV-positive patients with fever in virus-endemic areas developed hearing loss, and 17.6% of them had permanent hearing loss, while among those who had been exposed to LASV and were antibody-positive, 17.6% developed SNHL; 81.2% of the residents with sudden hearing loss in the study area were seropositive for LASV, and 71.9% of them had at least one ear with profound hearing loss. The incidence of SNHL caused by Lassa fever is higher than previously recognized [220]. Yun et al. established a mouse model of LASV infection to study hearing loss. Survival mice infected with human LASV isolate developed permanent hearing loss. The cochlear hair cells were slightly damaged, and the spiral ganglion cells of the auditory nerve were significantly degraded [221]. Maruyama et al. have shown that CD4 T cells play an essential role in LASV-induced hearing loss, while CD8 T cells are critical in the pathogenicity of the acute phase of LASV infection [222]. 

Ribavirin has been considered the standard treatment for many years [223]. Ribavirin is an ototoxic drug. However, the prognosis and progression of SNHL are not related to ribavirin use, while the persistence of SNHL despite ribavirin treatment also suggests that reduced viremia cannot slow the progress of HL [219]. These manifestations indicate that the pathogenesis of SNHL is not directly caused by LASV infection but is immune-mediated [220,224]. However, this mechanism has not yet been experimentally proven. In a study by Ibekwe et al., Lassa fever patients who developed SNHL were treated with steroids, hyperbaric oxygen, labyrinth vasodilators, and vitamin supplements and given hearing aids but experienced no improvement, which suggests that LASV-induced SNHL can cause extensive nerve damage requiring cochlear implant-based correction [225]. More research is still needed to develop effective treatment methods. 

## 11. Influenza A Virus

Influenza A virus belongs to family *Orthomyxoviridae* and has a segmented negative-sense, single-stranded RNA genome. The main symptoms of infection are fever, cough, sore throat, and diarrhea and other gastrointestinal symptoms [226]. 

Blum et al. reported, for the first time, that a 73-year-old male patient developed hearing loss after being infected with the H1N1 variant of influenza A. The patient was treated with high-dose prednisolone (60 mg per day). After 24 h, hearing improved, the sensation of fullness was reduced, and the patient felt well and did not complain of discomfort [227]. Alsanosi et al. reported two cases of bilateral hearing loss after H1N1 infection. One involved a two-year-old girl with normal hearing and language development commensurate with age before infection. She was treated with oseltamivir and developed severe bilateral SNHL two months after the infection. Language development was stable after wearing hearing aids for three months. The other case was of a three-month-old boy who passed hearing screening at birth, received oseltamivir treatment after H1N1 infection, and showed no abnormalities in an imaging examination, but stopped responding to sound and showed profound bilateral SNHL; he was treated with hearing aids [228]. Huang at al reported on a girl experiencing sudden sensorineural deafness after receiving the H1N1 vaccine. The girl was 17 years old and experienced hearing loss 14 h after receiving the H1N1 vaccine. She also experienced mild dizziness, nausea, and bilateral tinnitus but had no dizziness, vomiting, or other symptoms. PTA showed a hearing loss of 55 dB in both ears. Tympanography showed normal compliance of both eardrum membranes. Brain MRI showed no pathological findings. Her hearing was restored to 30 dB after treatment with corticosteroids, dextran, and vitamin B complex [229]. 

H1N1 is sensitive to neuraminidase inhibitors, and mainly oseltamivir or zanamivir are used for antiviral therapy, in addition to supportive therapy [230]. According to current reports, antiviral therapy is ineffective against hearing loss caused by H1N1, and prednisolone may improve hearing symptoms [227,228], suggesting that inflammation may be involved in the process.

## 12. Mumps Virus

Mumps virus is an enveloped single-stranded virus belonging to the family *Paramyxoviridae*. Humans are the only reservoir for the mumps virus. The virus is mainly transmitted through droplets. The virus first proliferates in the epithelial cells of the nose or upper respiratory tract and then enters the blood to cause viremia, which spreads to the salivary glands and other organs [231]. This causes mumps with parotid gland swelling and pain as the main symptoms, more commonly in children and adolescents, which may be complicated by aseptic meningitis and encephalitis, as well as orchitis and oophoritis [231]. The incubation period of the Mumps virus is two to four weeks [232]. The disease lasts about seven to twelve days, and lasting immunity can be achieved thereafter.

SNHL is a common complication of mumps [231], and the incidence is about 1/1000–20,000 cases of infection, and there is a clear age peak between the ages of five to nine [233,234]. Vuori et al. found that, among adult male soldiers, 4.4% of mumps patients had hearing loss, which mainly occurred at high frequencies and was reversible [235]. However, this sample did not include children, and the sample size was small, while other studies reported different results. A study by Morita et al. found that 94% of patients with hearing loss due to mumps developed unilateral SNHL, and the vast majority presented with profound hearing loss. Only one HL case (3.4%) treated with steroids showed a complete recovery [236]. In symptomatic patients with mumps infection, hearing loss is more likely to occur on the ipsilateral side if unilateral parotid swelling is present, or hearing loss is more likely to occur on the side with more severe swelling if both parotid glands are swollen [237]. In addition, studies have also found that patients with mumps virus infection may have vestibular symptoms, such as vertigo and tinnitus, and they appear on the same side as hearing loss [231,237].

Relevant studies have shown that, when the mumps virus is inoculated into mouse cerebrospinal fluid or immature cochlea, viral antigens can be observed in stria vascularis, vestibular membrane, and supporting cells [238]. Tanaka et al. used a different method to infect mice and observed the same results, but when the mumps virus was inoculated into the perilymph via the intralabyrinthine route, cochlear damage occurred mainly in the lower part of the cochlea, whereas intravascular inoculation of the virus had no related injuries found [239]. Lindsay et al. first reported the histopathology of the temporal bone in a patient with mumps-induced deafness, in which the consequence showed degeneration of the stria vascularis and tectorial membrane, as well as the organ of Corti in the middle and base regions, which the researchers hypothesized to be due to viremia affecting the stria vascularis, and then endolymphatic labyrinthitis caused hearing loss [240]. Similar results were also reported by Smith et al., who found alterations in the organ of Corti, but no corresponding pathological findings in the tectorial membrane and stria vascularis [241].

The clinical diagnosis of Mumps virus is based on the acute attack of the unilateral or bilateral parotid gland or other salivary gland swellings, which lasts for two days or more, and no other apparent cause is found [242]. Swelling is a typical symptom of mumps, but patients with the subclinical infection still require laboratory diagnosis. Laboratory diagnosis mainly includes virus isolation, nucleic acid detection, and serological analysis. 

There is currently no specific antiviral therapy for mumps, and interventions are mainly based on relieving clinical symptoms and treating complications. For the treatment of hearing loss, steroid hormones, vitamin B, and hyperbaric oxygen therapy may be ineffective in most patients. In the study of Morita et al., only one patient showed complete hearing recovery. While patients with total bilateral deafness received cochlear implants and achieved good results in speech perception, and a patient with unilateral deafness improved directional hearing by using audiphones [236]. Noda et al. reported two cases of bilateral mumps patients with cochlear implants. One patient had improved hearing, but the other had no change in speech perception. It is speculated that there may be lesions in the central nervous system [243]. For bilateral hearing loss caused by the mumps virus, a cochlear implant may improve hearing, but a careful evaluation of central involvement is required.

## 13. Measles Virus

Measles virus is a member of the genus *Paramyxoviridae*, a family of measles viruses and mumps. Measles is a highly contagious, acute infectious disease to which the population is universally susceptible, most commonly in infants, adults over 20 years of age, pregnant women, and immunocompromised or malnourished individuals, especially children who are vitamin A-deficient [244]. The Measles virus is mainly transmitted by respiratory droplets over short distances and can remain suspended in the air for 12 h [245].

Measles is an acute febrile disease associated with characteristic erythematous papules. The disease begins with fever and is usually accompanied by at least one of the symptoms of cough, rhinitis, and conjunctivitis. The virus can proliferate in the dermis and form a characteristic Koplik spot. Koplik spots usually appear as bluish-white spots, slightly raised with a diameter of approximately 2 to 3 mm on an erythematous base on the buccal mucosa opposite the first molar. The Koplik spot can serve as a characteristic sign and provide a clinical diagnosis of measles one to two days prior to the appearance of the rash. After three to four days of fever, the patient develops a characteristic rice bran-like rash that spreads throughout the body and lasts about one week [246]. Most children recover from measles without sequelae and acquire long-term immunity. Pneumonia secondary to viral infections may be the leading cause of measles-related complications and mortality [247]. Survivors of measles-related brain or spinal cord inflammation may experience permanent sequelae, including intellectual disability, seizures, motor abnormalities, and deafness [248].

It is estimated that measles infection accounted for 5–10% of all cases of severe bilateral SHNL before the introduction of the live measles vaccine [249]. Measles causes hearing loss ranging from typically bilateral model to profound SNHL. It was observed, in the temporal bone pathological specimens of measles-infected patients, that diffuse cochlear pathology with destruction or degeneration of the organ of Corti, stria, and cochlear neurons is typical. The most severe changes are usually found in the basal turn. In some specimens, endomorphic hydrops can be seen [250]. In addition, most observational studies have detected measles virus RNA in the otosclerotic stapes by different methods. As such, measles virus may be a causative agent of otosclerosis, and patients may develop conductive deafness as a result [251]. Measles is associated with a high incidence rate of otitis media. Measles and other viruses have also been detected in some patients’ otitis media effusion.

The most common laboratory method to confirm measles virus infection is the detection of measles virus-specific IgM in serum or plasma. However, measles virus-specific IgM may only be detected four or more days after the onset of the rash, leading to false-negative results if samples are collected too early [252]. Confirming of measles virus infection can also be achieved by RT-PCR detection of viral RNA using the throat, nasal, nasopharyngeal, and urine samples before IgM is detected [246].

Live attenuated measles virus vaccine has the potential to cause HL. There is one case suffer from HL per 6–8 million doses based on the annual use of MMR vaccine [253]. The hearing loss caused by vaccination can be bilateral or unilateral, and the severity varies. However, MMR vaccination remains the most effective method of measles prevention. The first dose is at the age of twelve to fifteen months, and the second dose is at the age of four to six years.

After measles infection, adults or children with normal immunity should be given supportive therapy to correct or prevent dehydration and nutritional deficiencies. At the same time, secondary bacterial infections should be promptly detected and treated, and patients should be provided with vitamin A. Antibiotics are applicable when there is clinical evidence of infection, but preventive use of antibiotics is not recommended [246]. Patients with measles infection induced hearing loss can wear hearing aids or cochlear implants. For patients infected with measles induced otosclerosis, stapedotomy can be performed to treat their conductive loss. Wearing hearing aids and cochlear implants is also helpful [254].

## 14. Lymphocytic Choriomeningitis Mammarenavirus

*Lymphocytic choriomeningitis mammarenavirus* (LCMV) belong to the *Arenaviridea* family *Mammavirus*. Human infection occurs through mucosal exposure to aerosols contaminated with rodent excreta, direct contact with rodents, or through rodent bites [255]; organ transplantation [256] and vertical mother-to-infant transmission [257] are also possible ways. Symptomatic-acquired LCMV infection usually presents with a biphasic course; the initial symptoms are nonspecific and include fever, headache, malaise, myalgia, anorexia, nausea, and vomiting. After temporary improvement, a second stage occurs, manifested by CNS symptoms, such as headache, photophobia, vomiting, and neck stiffness [258]. In rare cases, patients develop aseptic meningitis or meningoencephalitis, but usually recover without sequelae [259].

Fetal LCMV infection may occur during symptomatic maternal viremia, mainly in the first and second trimesters of pregnancy, and LCMV has embryonic teratogenicity. Ninety percent of the infants surveyed by Barton et al. developed chorioretinitis, 91.7% of infants who had undergone imaging had hydrocephalus or periventricular calcification, and 86% had long-term neurological abnormalities during follow-up, including microcephaly, moderate to severe mental retardation, seizures, and visual impairment. Although the incidence of hearing impairment is low, such cases are still reported [260]. 

Currently, the primary detection method of LCMV is the antibody titer of acute and convalescent serum samples, while detecting specific IgM in blood and cerebrospinal fluid is of diagnostic value [261]. Both enzyme-linked immunosorbent assay (EIA) and immunofluorescence assay (IFA) can detect IgM and IgG antibodies. For children with congenital infection, serological detection of LCMV could include the titers of IgM and IgG in maternal and infant serum samples [258]. 

Antiviral therapy is limited. Ribavirin has been proven effective against LCMV in vitro, but there is still a lack of solid evidence treating of human LCMV infection [262]. Farravir is effective in mouse models and can inhibit virus replication to an undetectable level [263]. Umifenovir is an indole carboxylic acid that inhibits the replication of LCMV in vitro [264].

## 15. Toscana Virus

Toscana virus (TOSV), belonging to the genus *Phlebovirus*, family *Phenuiviridae*, order *Bunyavirales*, is a kind of arbovirus closely related to the distribution of insect vectors [265]. TOSV is prevalent in the Mediterranean region and is the most common cause of meningitis locally between May and October. TOSV is the only neurotropic virus transmitted by sand flies, which can cause aseptic acute encephalitis, meningitis, and meningoencephalitis, but like other arboviruses, most people are asymptomatic when infected with TOSV. In Spain, a 63-year-old patient was reported to have developed hearing loss, fever, and meningitis, which was relieved except for tinnitus and bilateral SNHL one year later [266]. An 82-year-old male patient who returned from Italy to the United States was diagnosed with Toscana virus encephalitis and developed fever, severe weakness, and hearing loss [267].

Although the distribution of TOSV is concentrated, there are still imported cases in other areas. The key to diagnosis is to consider the patient’s symptoms and sojourn history. TOSV infection can be diagnosed directly through identification of the virus by molecular detection and/or serologically through the detection of specific antibodies. There are already several commercial test kits available to detect TOSV. Viruses can be isolated from blood or cerebrospinal fluid during the acute phase of the disease, but detection of the TOSV genome using real-time RT-PCR is currently the reference test for diagnostic purposes [265].

## 16. Discussion

Researchers believe that the three mechanisms of hearing loss caused by viral infection are: neuritis caused by viral involvement of cochlear nerves, cochlear inflammation caused by viruses, and stress response caused by cross-reaction of inner ear antigens to viral infection [268]. 

Prevention is essential to reduce the incidence of viral infections and complications. Safe, effective, and inexpensive vaccines are an essential means of controlling the spread of the virus. However, in many less developed countries, the vaccination rate is still low, large-scale herd immunity cannot be formed, and small-scale or large-scale outbreaks remain. Even in developed countries, there are often imported cases. In addition, vaccine prevention and control are difficult to achieve for sudden outbreaks, large-scale epidemics, and rapidly mutating viruses, such as SARS-CoV-2. Therefore, there is a need to develop drug treatment regimens for virus-induced hearing loss.

Unfortunately, the effect of antiviral therapy on virus-associated congenital hearing loss is still controversial. After cessation of antiviral therapy, viral replication may rebound with hearing loss. In the actual diagnosis and treatment of SSNHL patients, virus screening or antiviral treatment is usually not considered. For congenital deafness, it is difficult to determine whether the infant’s hearing loss is caused by congenital CMV infection, as CMV screening is not included in the newborn hearing screening. Delayed testing can only speculate whether the newborn has been infected with CMV, but cannot prove that CMV infection caused hearing loss, which makes it difficult for doctors to choose whether to use antiviral therapy or not and may miss the best treatment opportunity. In addition, it is difficult to judge whether the virus content in the blood can represent the virus content in the cochlea and predict the severity of the hearing loss, as the virus content in each system tissue of an infected person is different. More efficient tests are needed to assess the relationship between viral infection and hearing loss severity. 

This review summarizes the viruses reported to cause hearing loss, including their epidemiological characteristics, hearing phenotypes, and possible pathogenesis. Further research is needed to elucidate the precise etiopathogenesis of virus-induced hearing loss to better understand of the disease.

## Figures and Tables

**Table 1:** Viruses that may cause hearing loss.

Genbank Common Name	Genome Type	Genome Size	Order	Family	Genus
Cytomegalovirus	dsDNA	240 kb	*Herpesvirales*	*Herpesviridae*	*Cytomegalovirus*
Herpes simplex virus	dsDNA	150 kb	*Herpesvirales*	*Herpesviridae*	*Simplexvirus*
Varicella-zoster virus	dsDNA	120–130 kb	*Herpesvirales*	*Herpesviridae*	*Varicellovirus*
Epstein-Barr virus	dsDNA	172 kb	*Herpesvirales*	*Herpesviridae*	*Lymphocryptovirus*
Severe acute respiratory syndrome coronavirus 2	+ssRNA	29.9 kb	*Nidovirales*	*Coronaviridae*	*Betacoronavirus*
Hepatitis B virus	dsDNA	3.2 kb	*Blubervirales*	*Hepadnaviridae*	*Orthohepadnavirus*
Human immunodeficiency virus	+ssRNA	9.18 kb	*Ortervirales*	*Retroviridae*	*Lentivirus*
Rubella virus	+ssRNA	9.7 kb	*Hepelivirales*	*Matonaviridae*	*Rubivirus*
Zika virus	+ssRNA	10.8 kb	*Amarillovirales*	*Flaviviridae*	*Flavivirus*
West Nile virus	+ssRNA	10–11 kb	*Amarillovirales*	*Flaviviridae*	*Flavivirus*
Human enterovirus	+ssRNA	7.4 kb	*Picornavirales*	*Picornaviridae*	*Enterovirus*
Lassa virus	−ssRNA	10.7 kb	*Bunyavirales*	*Arenaviridae*	*Mammarenavirus*
Influenza virus	−ssRNA	13.6 kb	*Articulavirales*	*Orthomyxoviridae*	
Mumps orthorubulavirus	−ssRNA	15.3 kb	*Mononegavirales*	*Paramyxoviridae*	*Orthorubulavirus*
Measles virus	−ssRNA	16 kb	*Mononegavirales*	*Paramyxoviridae*	*Morbillivirus*
Lymphocytic choriomeningitis mammarenavirus	−ssRNA	11 kb	*Bunyavirales*	*Arenaviridae*	*Mammarenavirus*
Toscana virus	−ssRNA	12.5 kb	*Bunyavirales*	*Phenuiviridae*	*Phlebovirus*

The information in Table 1 comes from the National Library of Medicine (National Center for Biotechnology Information (nih.gov)).
